# Supplementary material for: Towards rapid intraoperative axial localization of spinal cord ischemia with epidural diffuse correlation monitoring
Source: PLoS One. 2021 May 10;16(5):e0251271. doi: 10.1371/journal.pone.0251271 (PMC8109798; doi:10.1371/journal.pone.0251271)
Supplement: S1 Table — Each inflation-site pair is labeled with the radiographically determined location of the aortic occlusion balloon (is the balloon cephalad, true/false). (PDF) [file pone.0251271.s001.pdf]

S1 Table:

Tabulation of changes in relative blood flow (rBFI %) during serial inflations (Inflation #) at three sites (Site #, 1 is most cephalad) in each of five pigs (pig #). Each inflation-site pair is labeled with the radiographically determined location of the aortic occlusion balloon (is the balloon cephalad, true/false).

Pig #	Site #	Inflation #	Balloon cephalad?	rBFI [%]
1	1	1	TRUE	-60.2
1	2	1	FALSE	-46.9
1	3	1	FALSE	-69.9
1	1	2	FALSE	-32.1
1	2	2	FALSE	-62.1
1	3	2	FALSE	-61.1
1	1	3	TRUE	-63.8
1	2	3	FALSE	-67.7
1	3	3	FALSE	-74.9
1	1	4	TRUE	-29.4
1	2	4	TRUE	42.2
1	3	4	FALSE	-36.5
1	1	5	TRUE	18.8
1	3	5	FALSE	-71.6
1	1	6	TRUE	-28.2
1	2	6	FALSE	-55.5
1	3	6	FALSE	-82.8
1	1	7	FALSE	-35.6
1	2	7	FALSE	-71.1
1	3	7	FALSE	-45.5
2	2	1	FALSE	-59.7
2	3	1	FALSE	-61.7
2	1	2	TRUE	14.1
2	2	2	FALSE	32.0
2	3	2	FALSE	-52.1
2	1	3	TRUE	1.3
2	2	3	TRUE	53.8
2	3	3	FALSE	-42.9
2	1	4	TRUE	41.7
2	2	4	FALSE	-58.4
2	3	4	FALSE	-78.1
2	1	5	TRUE	3.4
2	2	5	TRUE	9.1
2	3	5	TRUE	0.9
2	1	6	TRUE	-2.0
2	2	6	TRUE	15.4
2	3	6	TRUE	18.9
3	1	1	FALSE	-45.5
3	2	1	FALSE	-59.3
3	3	1	FALSE	-79.0
3	1	2	TRUE	17.7
3	2	2	FALSE	-18.7
3	3	2	FALSE	-27.3
3	1	3	TRUE	45.6
3	2	3	TRUE	122.1
3	3	3	FALSE	38.5
3	1	4	TRUE	10.2
3	2	4	TRUE	-18.7
3	3	4	TRUE	12.1
3	1	5	FALSE	-73.5
3	2	5	FALSE	-68.9
3	3	5	FALSE	-70.5
4	1	1	FALSE	-39.3
4	2	1	FALSE	-71.9
4	3	1	FALSE	-76.5
4	1	2	TRUE	31.5
4	2	2	FALSE	-70.0
4	3	2	FALSE	-77.3
4	1	3	TRUE	2.2
4	2	3	TRUE	36.5
4	3	3	FALSE	-57.2
4	1	4	TRUE	-9.7
4	2	4	TRUE	-14.4
4	3	4	TRUE	26.3
4	1	5	FALSE	-58.0
4	2	5	FALSE	-81.1
4	3	5	FALSE	-85.0
4	1	6	TRUE	52.9
4	2	6	FALSE	-77.3
4	3	6	FALSE	64.2
5	1	1	FALSE	-74.3
5	2	1	FALSE	-84.7
5	3	1	FALSE	-77.0
5	1	2	TRUE	-38.3
5	2	2	FALSE	-81.9
5	3	2	FALSE	-73.5
5	1	3	TRUE	-2.3
5	2	3	TRUE	-14.1
5	3	3	FALSE	-62.4
5	1	4	TRUE	16.3
5	2	4	TRUE	9.3
5	3	4	TRUE	16.0
5	1	5	TRUE	16.1
5	2	5	TRUE	17.2
5	3	5	TRUE	16.3
